# Supplementary material for: A large language model for electronic health records
Source: NPJ Digit Med. 2022 Dec 26;5:194. doi: 10.1038/s41746-022-00742-2 (PMC9792464; doi:10.1038/s41746-022-00742-2)
Supplement: Supplementary file 1 — SUPPLEMENTAL MATERIAL [file 41746_2022_742_MOESM1_ESM.pdf]

Supplementary Information

Supplementary Table 1. Summary of patients from 2011-2021 at UF Health.

Demographics	Sub-categories	Descriptive Statistics (N=2,476,628)	Percentage of the cohort (%)
Age (As of September 2022)	<18	338,676	13.67%
	18 - 65	1,439,493	58.12%
	>65	698,126	28.19%
	Unknown	333	0.01%
Gender	Female	1,307,511	52.79%
	Male	1,168,746	47.19%
	Unknown	371	0.01%
Ethnicity	Hispanic	123,608	4.99%
	Non-Hispanic	1,495,019	60.37%
	Unknown	858,001	34.64%
Race	Black	382,742	15.45%
	White	1,079,793	43.60%
	Other	187,161	7.56%
	Unknown	826,932	33.39%

Supplementary Table 2. Distribution of clinical notes among 126 clinical departments.

Department/Specialty	Percentage
Family Medicine	18.61%
Emergency Medicine	8.35%
Primary Care	6.20%
Internal Medicine	5.25%
Orthopedics	3.72%
Hospitalist	3.54%
Radiology	3.00%
Psychiatry	2.85%
Obstetrics & Gynecology	2.81%
Neurosurgery	2.55%
Surgery	2.55%
Neurology	2.32%
Obstetrics	2.28%
Pediatrics	2.25%
Cardiology	1.88%
Gastroenterology	1.72%

General Surgery	1.57%
Urology	1.40%
Oncology	1.31%
Rehabilitation	1.23%
Trauma-Acute Care	1.12%
Neonatology	1.06%
Ophthalmology	1.01%
Otolaryngology	0.93%
Medicine Orange	0.91%
Pediatric Surgery	0.87%
Heart Fail/Tx C	0.83%
Pediatric Primary Care	0.81%
General Internal Medicine	0.77%
Endocrinology	0.77%
Dermatology	0.76%
Nephrology	0.71%
Lab	0.71%
Intensive Care	0.63%
Thoracic Cardiovascular	0.62%
Pediatric General Medicine	0.59%
Cardiology/Cardiovascular Disease	0.55%
Gynecology	0.53%
Pulmonary	0.51%
Hematology and Oncology	0.51%
Rheumatology	0.46%
Vascular	0.42%
Hematology	0.40%
Pediatric Intensive Care	0.40%
Hepatology	0.38%
Pain Management	0.38%
Pediatric Hematology/Oncology	0.35%
Pulmonology	0.33%
Surgical Intensive Care	0.32%
Oral and Maxillofacial Surgery	0.30%
Pediatric Cardiology	0.29%
Pediatric Newborn	0.27%
Pediatric Gastroenterology	0.27%
Vascular Surgery	0.26%
Pediatric Critical Care Medicine	0.26%
Pediatric Endocrinology	0.26%
Plastic Surgery	0.25%

Pediatric Neurology	0.22%
Surgery - Pancreaticobiliary	0.21%
Pediatric Rheumatology	0.21%
Radiation Oncology	0.18%
Cardio Teaching	0.18%
Burn Surgery / Burn Care	0.18%
Anesthesiology	0.17%
Sleep Center	0.16%
Pediatric Infectious Disease	0.14%
Lung Transplant	0.13%
Psychology	0.13%
Pediatric Pulmonology	0.13%
Geriatric Medicine	0.13%
Infectious Diseases	0.13%
Infusion Therapy	0.12%
Unknown Specialty	0.11%
Child/Adolescent Psychiatry	0.11%
Pediatric Nephrology	0.10%
Interventional Cardiology	0.10%
Dialysis	0.09%
Cardiothoracic Surgery	0.08%
Pediatric Allergy	0.08%
Respiratory Therapy	0.07%
Pediatric Urology	0.07%
Allergy	0.06%
Pediatric Genetics	0.06%
Pediatric Neurosurgery	0.06%
Podiatry	0.05%
Surgical Oncology	0.04%
Pediatric Rehabilitation	0.04%
Bariatrics	0.03%
Physical Medicine and Rehab	0.02%
Pathology	0.02%
Pharmacy	0.02%
Nuclear Medicine	0.02%
Nutrition	0.01%
Behavioral Health	0.01%
Urgent Care	0.01%
Integrative Medicine	0.01%
Mycobacteriology	0.01%
Occupational Medicine	0.01%

Sports Medicine	0.01%
Employee Health	0.01%
Critical Care Medicine	0.01%
Audiology	<0.01%
Diabetes Services	<0.01%
Gerontology	<0.01%
Interventional Radiology	<0.01%
Developmental	<0.01%
Optometry	<0.01%
Care Coordination	<0.01%
Critical Care/Pulmonology	<0.01%
Cardiac Rehabilitation	<0.01%
Dentistry	<0.01%
Child Heart Center	<0.01%
Orthopedic Surgery	<0.01%
Home Health Services	<0.01%
Pediatric Cardiothoracic Surgery	<0.01%
Surgery - MIS/Bariatric	<0.01%
Colon and Rectal Surgery	<0.01%
Social Services	<0.01%
Transplant Surgery	<0.01%
Pediatric Oncology	<0.01%
Surgery-Brst/Mela/Sarc/Endoc	<0.01%
Breast Clinic / Breast Center	<0.01%
Pastoral Care	<0.01%
Medical Records	<0.01%
Immunology	<0.01%
Orthotics	<0.01%

Supplementary Table 3. Distribution of clinical notes among 101 note types.

**Note Type**	**Percentage**
Progress Notes	23.91%
Telephone Encounter	12.49%
Plan of Care	4.40%
ED Notes	3.84%
After Visit Summary	2.56%
Procedures	2.20%
Letter	2.20%
Problem Overview	1.28%
Consults	1.14%

ED Provider Notes	0.97%
H&P	0.95%
Patient Instructions	0.94%
Cosign with Attestation Text	0.90%
Discharge Summary	0.81%
Op Note	0.59%
Psych Progress Note	0.55%
ED Attestation Note	0.52%
ED AVS Snapshot	0.48%
Psych Nurses Notes	0.40%
Miscellaneous	0.34%
General	0.32%
ED Triage Notes	0.30%
Addendum Note	0.29%
IP AVS Snapshot	0.28%
Anesthesia Preprocedure Evaluation	0.26%
Anesthesia Postprocedure Evaluation	0.23%
Assessment & Plan Note	0.18%
Immediate Post-Operative Note	0.16%
Plan of Care Update	0.14%
Interval H&P Note	0.11%
Discharge Instr - AVS First Page	0.11%
Case Attestation Note	0.10%
H&P (View-Only)	0.10%
Sticky Note	0.09%
Medical Student	0.09%
Coding Query	0.08%
Anesthesia Procedure Notes	0.08%
Nursing Post-Procedure Note	0.07%
Patient Care Transfer Note	0.07%
Lactation Note	0.05%
Psych H&P	0.05%
ANE Pre-op Evaluation	0.04%
Psych Procedure Note	0.04%
ANE Post-op Evaluation	0.03%
ED Continuation of Care Note	0.03%
Initial Plan of Care	0.03%
Team Conference	0.03%
Nursing Intra-Procedure Note	0.02%

Psych DC Summary	0.02%
Outpatient Student	0.02%
Medical Screening Note	0.02%
Psych	0.02%
Care Coordination	0.02%
L&D Delivery Note	0.02%
Psych Consult	0.02%
Psych Plan of Care	0.02%
Treatment Plan	0.01%
Sedation Documentation	0.01%
ED CDU H&P	0.01%
Code Documentation	0.01%
Post Admission Assessment	0.01%
ED CDU Note	0.01%
OR PostOp	0.01%
Nursing Pre-Procedure Note	0.01%
Decision for Case	0.01%
Psych Team Conference	0.01%
History Event Details	0.01%
Treatment Summary	0.01%
OR Anesthesia	0.01%
Patient Care Conference	0.01%
OR PreOp	<0.01%
Progress Note	<0.01%
ED CDU Attestation Note	<0.01%
Significant Event	<0.01%
Pulm Lab Procedure Note	<0.01%
Discharge Instr - Appointments	<0.01%
Discharge Instr - Activity	<0.01%
Infection Case	<0.01%
Code Status and Advance Directives	<0.01%
Discharge Instr - Diet	<0.01%
Discharge Instr - Other Orders	<0.01%
Psych Interval H&P	<0.01%
Nursing Pre-Procedure PAT Note	<0.01%
OR Surgeon	<0.01%
Emergent Intervention Note	<0.01%
Radiation Planning Notes	<0.01%
Radiation Completion Notes	<0.01%

Ethics Committee Note	<0.01%
Discharge Instr - Lab	<0.01%
History Overview	<0.01%
ACP (Advance Care Planning)	<0.01%
Transplant	<0.01%
Consult Note	<0.01%
Subjective & Objective	<0.01%
OR Nursing	<0.01%
Psych Evaluation	<0.01%
Discharge Instr - Supplementary Instructions	<0.01%
H&P Note	<0.01%
Procedure Note	<0.01%
Initial Assessments	<0.01%
Communication Body	<0.01%
Unspecified	28.68%
