# Supplementary material for: A large language model for electronic health records
Source: NPJ Digit Med. 2022 Dec 26;5:194. doi: 10.1038/s41746-022-00742-2 (PMC9792464; doi:10.1038/s41746-022-00742-2)
Supplement: Supplementary file 2 — Reporting Summary [file 41746_2022_742_MOESM2_ESM.pdf]

nature portfolio

Corresponding author(s): Yonghui Wu

Last updated by author(s): 11/02/2022

Reporting Summary

Nature Portfolio wishes to improve the reproducibility of the work that we publish. This form provides structure for consistency and transparency in reporting. For further information on Nature Portfolio policies, see our Editorial Policies and the Editorial Policy Checklist.

Statistics

For all statistical analyses, confirm that the following items are present in the figure legend, table legend, main text, or Methods section.

n/a	Confirmed	
☒	☐	The exact sample size (*n*) for each experimental group/condition, given as a discrete number and unit of measurement
☒	☐	A statement on whether measurements were taken from distinct samples or whether the same sample was measured repeatedly
☒	☐	The statistical test(s) used AND whether they are one- or two-sided<br>*Only common tests should be described solely by name; describe more complex techniques in the Methods section.*
☒	☐	A description of all covariates tested
☒	☐	A description of any assumptions or corrections, such as tests of normality and adjustment for multiple comparisons
☒	☐	A full description of the statistical parameters including central tendency (e.g. means) or other basic estimates (e.g. regression coefficient) AND variation (e.g. standard deviation) or associated estimates of uncertainty (e.g. confidence intervals)
☒	☐	For null hypothesis testing, the test statistic (e.g. *F*, *t*, *r*) with confidence intervals, effect sizes, degrees of freedom and *P* value noted<br>*Give P values as exact values whenever suitable.*
☒	☐	For Bayesian analysis, information on the choice of priors and Markov chain Monte Carlo settings
☒	☐	For hierarchical and complex designs, identification of the appropriate level for tests and full reporting of outcomes
☒	☐	Estimates of effect sizes (e.g. Cohen's *d*, Pearson's *r*), indicating how they were calculated

Our web collection on statistics for biologists contains articles on many of the points above.

Software and code

Policy information about availability of computer code

Data collection	No commercial software were used in data collection. All open-source and custom codes were provided in Github
Data analysis	No commercial software were used in data analysis. All open-source and custom codes were provided in Github

For manuscripts utilizing custom algorithms or software that are central to the research but not yet described in published literature, software must be made available to editors and reviewers. We strongly encourage code deposition in a community repository (e.g. GitHub). See the Nature Portfolio guidelines for submitting code & software for further information.

Data

Policy information about availability of data

All manuscripts must include a data availability statement. This statement should provide the following information, where applicable:

- Accession codes, unique identifiers, or web links for publicly available datasets
- A description of any restrictions on data availability
- For clinical datasets or third party data, please ensure that the statement adheres to our policy

All benchmark datasets that support the findings of this study are available from the official websites of natural language processing challenges with Data Use Agreements. Detailed links are provided in the "Data availability" section. UF Health IDR clinical notes are not open to the public due to patient privacy information; we provided detailed data distribution in the "Results" section.

Human research participants

Policy information about studies involving human research participants and Sex and Gender in Research.

Reporting on sex and gender	This study used the biological sex captured in UF Health Epic system. The findings apply to both sexes. This study focuses on the medical language documented in clinical narratives; we reported the overall proportion and number of patients for each sex group, the individual level data was not used. This study was approved by the UF Institutional Review Board (IRB202100049)
Population characteristics	Population characteristics were provided in the Result section.
Recruitment	This study has no recruitment of subjects, this is a retrospective cohort study using existing data.
Ethics oversight	This study was approved by the UF Institutional Review Board (IRB202100049)

Note that full information on the approval of the study protocol must also be provided in the manuscript.

Field-specific reporting

Please select the one below that is the best fit for your research. If you are not sure, read the appropriate sections before making your selection.

☐ Life sciences ☒ Behavioural & social sciences ☐ Ecological, evolutionary & environmental sciences

For a reference copy of the document with all sections, see nature.com/documents/nr-reporting-summary-flat.pdf

Behavioural & social sciences study design

All studies must disclose on these points even when the disclosure is negative.

Study description	We developed from scratch a large clinical language model – GatorTron – using >90 billion words of text (including >82 billion words of de-identified clinical text)
Research sample	A total number of 290,482,002 clinical notes from 2,476,628 patients were extracted from the UF Health Integrated Data Repository (IDR)
Sampling strategy	We collected all clinical notes created from 2011-2021 at the University of Florida Health.
Data collection	We extracted clinical notes from the UF Health Integrated Data Repository (IDR), the enterprise data warehouse of the UF Health system. All the notes were de-identified using a computer algorithm. The protected health information defined by HIPAA were removed from the notes.
Timing	From 2011 to 2021.
Data exclusions	NA
Non-participation	NA - no recruitment needed, it's a retrospective study.
Randomization	NA - no recruitment needed, it's a retrospective study.

Reporting for specific materials, systems and methods

We require information from authors about some types of materials, experimental systems and methods used in many studies. Here, indicate whether each material, system or method listed is relevant to your study. If you are not sure if a list item applies to your research, read the appropriate section before selecting a response.

Materials & experimental systems

n/a	Involved in the study	
☒	☐	Antibodies
☒	☐	Eukaryotic cell lines
☒	☐	Palaeontology and archaeology
☒	☐	Animals and other organisms
☐	☒	Clinical data
☒	☐	Dual use research of concern

Methods

n/a	Involved in the study	
☒	☐	ChIP-seq
☒	☐	Flow cytometry
☒	☐	MRI-based neuroimaging

Clinical data

Policy information about clinical studies

All manuscripts should comply with the ICMJE guidelines for publication of clinical research and a completed CONSORT checklist must be included with all submissions.

Clinical trial registration	NA - This is not a clinical trial.
Study protocol	NA - This is not a clinical trial.
Data collection	This is a retrospective study using clinical notes already captured in the electronic health record system from 2011 to 2021.
Outcomes	NA - This is not a clinical trial.
